# Floral symmetry: the geometry of plant reproduction

**DOI:** 10.1042/ETLS20210270

**Published:** 2022-08-22

**Authors:** Yuxiang Jiang, Laila Moubayidin

**Affiliations:** Department of Cell and Developmental Biology, John Innes Centre, NR4 7UH Norwich, U.K.

**Keywords:** bilateral symmetry, evolution, flowers, gynoecium, radial symmetry, symmetry transition

## Abstract

The flower is an astonishing innovation that arose during plant evolution allowing flowering plants — also known as angiosperms — to dominate life on earth in a relatively short period of geological time. Flowers are formed from secondary meristems by co-ordinated differentiation of flower organs, such as sepals, petals, stamens, and carpels. The position, number and morphology of these flower organs impose a geometrical pattern — or symmetry type — within the flower which is a trait tightly connected to successful reproduction.

During evolution, flower symmetry switched from the ancestral poly-symmetric (radial symmetry) to the mono-symmetric (bilateral symmetry) type multiple times, including numerous reversals, with these events linked to co-evolution with pollinators and reproductive strategies.

In this review, we introduce the diversity of flower symmetry, trace its evolution in angiosperms, and highlight the conserved genetic basis underpinning symmetry control in flowers. Finally, we discuss the importance of building upon the concept of flower symmetry by looking at the mechanisms orchestrating symmetry within individual flower organs and summarise the current scenario on symmetry patterning of the female reproductive organ, the gynoecium, the ultimate flower structure presiding over fertilisation and seed production.

## Introduction

Since the earliest known multicellular organisms (Ediacaran biota) emerged ∼580–540 million years ago (mya), symmetric patterns of gene expression and tissue arrangement have supported the development of multicellular organs/organisms during evolution, allowing species to conquer new ecological niches and support their fitness. Thus, the origin and evolution of body symmetry, of which bilateral and radial symmetry are most common, has been a central question for all biologists [1,2].

Plants pioneered life on land and quickly adapted their lifestyle to a drier environment by evolving features such as a vascular system to support growth and long-distance transport, stomata to support photosynthesis and gas exchanges, roots for structural support and increased water and nutrient uptake, seeds to encase genetic material and last but not least, the flower to orchestrate reproduction.

Flowering plants, known as angiosperms, emerged relatively late in the fossil records (early Cretaceous, ca.140 mya) [3], but quickly radiated in several species and occupied the land. With more than 260 000 extant species, they represent ca. 90% of all living land plants today [4]. Their widespread colonisation over a relatively short evolutionary time frame initially puzzled Charles Darwin, as it argued against a more gradual and streamlined evolution he postulated in his famous ‘*Origin of species*' [5].

The key innovation at the basis of the huge diversification of angiosperms is indeed the flower: a reproductive structure presiding over efficient fertilisation, seed production, and fitness. One hundred and fifty years of intense studies, from Darwin to the modern evo-devo approach, have contributed elucidating the importance of flower symmetry for species radiation among flowering plants [6].

Two main types of flower symmetry are widely found in nature: *zygomorphy* (mono-symmetry, i.e. bilateral symmetry) and *actinomorphy* (poly-symmetry, i.e. radial symmetry). Extant basal angiosperms (magnoliids) mainly display actinomorphic flowers. Radially symmetric flowers are morphologically accessible from all directions by diverse pollinators, thus augmenting fitness in a non-competitive environment. Bilateral flowers appeared later, in the Upper Cretaceous (Turonian), and this key innovation is tightly linked to the evolution of new reproductive strategies, i.e. specific, and sometimes unique, plant–pollinator interactions [7,8].

Interestingly, symmetry types are not always fixed but can change, either during the development of the flower (ontogenesis), i.e. the early developmental stages display a different symmetry compared with the mature flower, or in evolution (phylogenesis) as demonstrated by the reversion of some mono-symmetric, bilaterally symmetric flowers back to radial symmetry [9].

In this review, we will provide an overview of the morphological diversity of floral symmetry, the molecular and developmental basis underpinning the evolution of symmetric flowers and the functional relevance of flower symmetry for successful pollination and reproduction. Lastly, we will highlight the latest advancements in symmetry establishment and transition within the female reproductive structure to discuss the importance of a general control of symmetry in plant organs.

## The diversity of flower morphology defines flower symmetry

A typical flower combines multiple organs such as sepals, petals, stamens and carpels, each presiding over a specific function carefully tailored for efficient plant reproduction. Flower organs are organised into whorls with sepals and petals located on the outermost whorls (calyx and corolla, respectively), and stamens and carpels positioned inwards (androecium and gynoecium whorls, respectively). The morphology, number and position of sepals and petals (collectively known as perianth) mainly determines the architecture of the flower, thus it has always been the major phenotype/trait defining flower symmetry [10]. The symmetric perianth architecture forms the ‘scaffold' of the flower, so changes in its morphology, including symmetry types, might have straightforward consequences in flower function, e.g. efficient reproduction.

**Figure 1.**
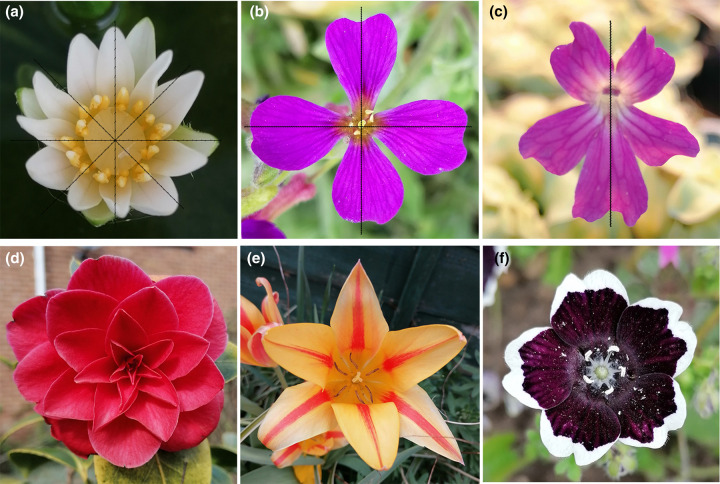
Flower symmetry types in angiosperms. Examples of (**a**) radial symmetry, (**b**) bi-radial symmetry and (**c**) bilateral symmetry in flowers, showing (**a**) multiple, (**b**) two, and (**c**) one axis/plane of symmetry. Based on the arrangement of flower organs radially symmetric flowers can be (**d**) *spiral*, in which floral parts are arranged spirally around the base, or thalamus, or (**e**,**f**) *cyclic*, in which all floral parts are arranged in concentric circles around the thalamus. Based on the number of floral organs in each distinct whorl, i.e. merosity, flowers can be classified in (**e**) trimerous, (**b**) tetramerous and (**f**) pentamerous flowers, in which the organs belonging to each whorl are consistently three, four, five (or multiples of that number), respectively. (**a**) Waterlily, *Nymphaea thermarum*; (**b**) *Aubrieta deltoidei*; (**c**) butterworts, *Pinguicula moranensis*; (**d**) *Camellia japonica*; (**e**) Asiatic lily, *Lilium auratum*; (**f**) *Nemophila Discoidalis*. Photos were taken by Yuxiang Jiang.

Based on the geometric feature, four types of flower symmetry can be distinguished:

- *Actinomorphic (radially symmetric)* flowers can be cut into identical halves by three or more planes of symmetry (Figure 1a). According to the number of flower organs present in the whorls (merosity, number of organs in a whorl), a specific rotation angle can be identified around which the body can be transformed in mathematical terms, to resemble the original shape. Thus, radially symmetric flowers can display from three to infinite folds of symmetry and include several species (Figure 1a,d–f).
- *Bi-/Di-Symmetric* flowers, can be divided in two identical halves by two axes of symmetry oriented perpendicularly to each other (90° angle): while the vertical axis defines the left–right halves, a second, horizontal axis virtually separates the adaxial/ventral (upper) half from the abaxial/dorsal (lower) half of the body (Figure 1b) [11]. Mathematically, rotation around the centre as well as reflection along both axes lead to equivalent images. Examples of bi-symmetric flowers include *Arabidopsis arenosa*.
- *Zygomorphic (bilaterally symmetric)* flowers, in which one single plane (or axis) of symmetry running through the centre of the body produces two equal halves that are mirror images of each other (Figure 1c) [11]. The left and right sides (along the medio-lateral body-axis) identified in this way can be transformed by a reflection along the vertical axis, and as such bilateral symmetry in biology can be described in mathematical terms. The most iconic example of the bilaterally symmetric flower is the snapdragon *Antirrhinum majus*.
- *Asymmetric* flowers cannot be divided into equal halves by any plane, e.g. the flower of *Canna indica*. Asymmetry can be generated by a reduction in the number of specific flower organs compared with those in other whorls, or by a break in bilateral symmetry generated by a deflection of an organ either to the left or right side, i.e. enantiostyly, the ‘style bending' of the *Solanum rostratum* gynoecium [12]. However, it is important to note that asymmetry does not equal to disorder; in fact, asymmetric flowers display higher-ordered complexity, in which breaking bilateral symmetry is often a strategy employed to co-ordinate pollen uptake and release by the pollinator visiting the flowers [6,13].

## The evolution of flower symmetry

In Cretaceous rocks (145–166 mya), fossils of flowering plants are believed to have appeared suddenly and in great diversity [3]. In 1879, Charles Darwin famously noted: ‘The rapid development as far as we can judge of all the higher plants within recent geological times is ‘an abominable mystery' [14]. Palaeobotanists, plant phytologists and geneticists have made huge efforts towards understanding Darwin's abominable mystery of angiosperm radiation [4,10,11,15,16].

**Figure 2.**
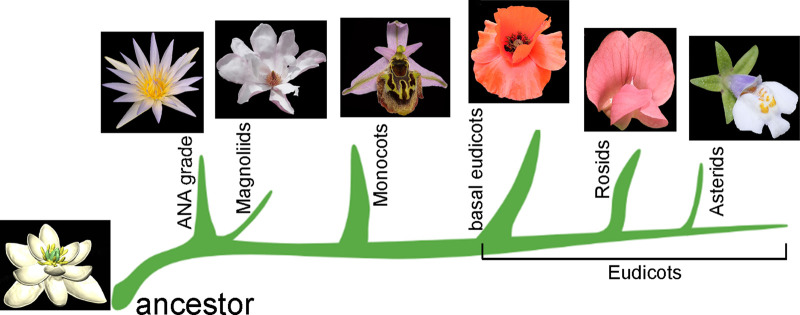
Simplified scheme of evolution of floral symmetry in Angiosperms. All images of representative flower symmetry patterns are adapted from the ‘Phylogeny of angiosperms poster' (http://www.plantgateway.com/poster/) following the APG IV classification [66] except the model-based prediction of the ancestor flower of angiosperms, which is adapted from Sauquet et al. [4] according to the article's Creative Commons Attribution 4.0 International License. Representative plants of main groups: ANA grade, Nymphaeaceae; Magnoliids, Magnoliaceae; Monocots, Orchidaecae; Basal eudicots, Papaveraceae; Rosids, Fabaceae; Asterids, Mazaceae.

Early diverging angiosperms (magnoliids) do no display bilateral flowers, and mono-symmetric flowers are rare among them. In line with this, a recent model-based study combined phylogeny and flower traits data to reconstruct the ancestral angiosperm flower, which showed radial symmetry (Figure 2) [4], providing new ways to explore the evolutionary hypothesis. However, in late-diverging eudicots, such as Rosids, Asterids and Fabaceae (legumes) and in lineages with a high density of species, such as Orchids, bilateral flowers are very common and often exhibit a high degree of complexity (Figure 2).

However, evolution is not always an unidirectional, straight forward process: in fact, while zygomorphy erose independently several times from actinomorphic ancestral flowers, reversal from zygomorphy to actinomorphy flowers has also been observed in several species [9,11].

Notably, the ‘pollination syndromes' concept have been postulated for a long time as the driving force behind zygomorphic flowers: a mutually beneficial co-evolution of plant–pollinator species that tightly links flower symmetry type, the ecology of visitor pollinators, and their fitness [17,18]. Fossil records demonstrate that the emergence of zygomorphic flowers coincides with the diversification of specialised insect pollinators [19], implying evolution of zygomorphy may facilitate plant–pollinator interactions over evolutionary time [20]. The astonishing precision by which pollen is placed on a pollinator's body during visits to zygomorphic flowers is exemplified by some species of orchids, in which flower shapes are so complex that they include highly specialised structures which only specific types of pollinators (insects and sometimes vertebrates) can access to feed, while passively supporting plant reproduction [21]. An extreme example of the complexity of bilateral flowers is the orchid genus *Ophrys* which produce blossoms that mimic the form and scent of a female wasp to attract/ deceive male wasps in search of a mating partner (Figure 2) [22,23]. Although the mutual benefit for both plants and animals is clear, the debate of who led this co-evolution, e.g. orchid or pollinators such as orchid bees, goes on [7].

## The genetic basis of floral symmetry

**Figure 3.**
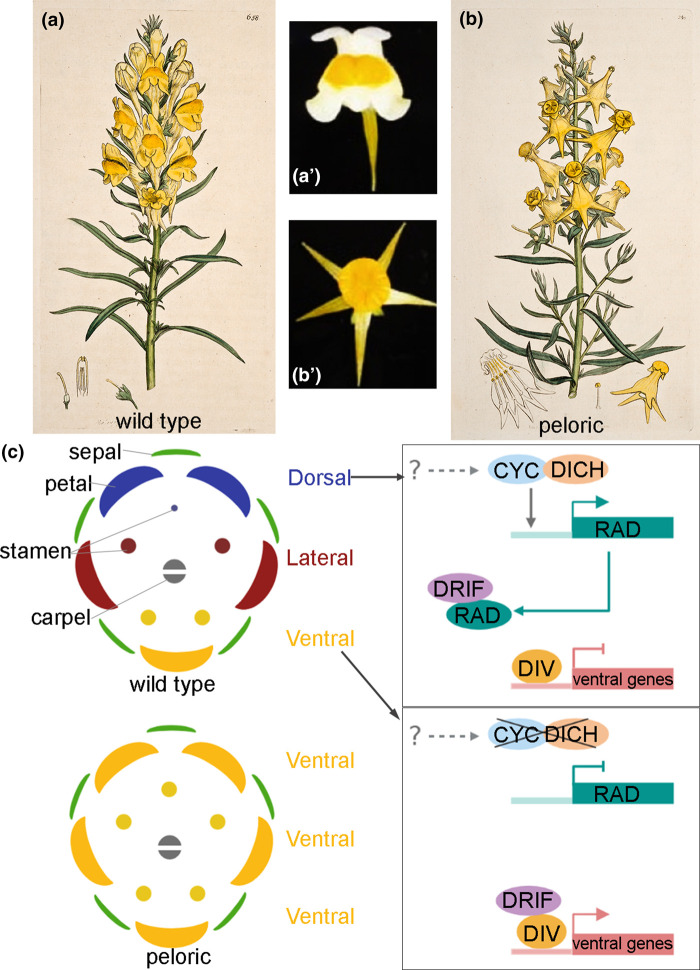
Genetic basis of floral symmetry. Example of (**a,b**) Inflorescence and (**a′,b′**) flower of (**a,a′**) wild-type and (**b,b′**) peloric mutant of toadflax (*Linaria vulgaris*) displaying bilateral and radial flower symmetry, respectively. (**a′,b′**) Adapted from Cubas et al. [26]. (**c**) Schematic flower diagrams and genetic regulation of flower symmetry and establishment of the dorsal–ventral body-axis. Key TFs from TPC family (CYC and DICH) activate *RAD* gene in the dorsal region. RAD sequesters DRIF, leaving DIV unable to promote ventralization. In the absence of TCPs or RAD, DRIF and DIV form heterodimers, promoting gene expression and ventral identity. Illustrations are created with Biorender.com.

The molecular basis of floral symmetry lies in a spatio-temporal orchestration of gene expression by specific transcription factors (TFs). In the bilateral flower of Plantaginaceae family members (including snapdragon and toadflax) dorsal and ventral domains display distinct morphological and molecular characteristics, i.e. dorsal–ventral (adaxial-abaxial) asymmetry (Figure 3a,b) [15,24–26]. For example, five petals are arranged along the adaxial–abaxial axis in three distinct positions — two dorsal, two lateral and one ventral — thanks to the activity of two teosinte BRANCHED1–CYCLOIDEA–PCF (TCP) domain-containing TFs, namely CYCLOIDEA (CYC) and its paralog DICHOTOMA (DICH), that control dorsal–ventral axis asymmetry (Figure 3c). Genetic studies of the *cyc dich* ‘peloric' mutant in snapdragon (*Antirrhinum majus*), which produce radially symmetric flowers with ectopic ventral identity, demonstrated their role in controlling flower bilateral symmetry by patterning the dorsal region and restricting the ventral domain [25–27]. Accordingly, the gene *CYC* is expressed at very early stages of flower development, in the dorsal domain of the flower meristem, where it affects growth rate and primordium initiation, thus establishing dorsoventral asymmetry [26]. Interestingly, also the morphological asymmetry displayed by individual petals is controlled by the same core mechanism, which is recruited and finetuned at different time points to reinforce the patterning of symmetry at different scales [25].

The expansion of TF families during plant evolution is positively correlated with flower complexity [27,28]. The MYB TF family is one of the most ancient found in animals and plants, and their role in flower development has been extensively studied in the past three decades. The MYB domain is the key feature of this protein family and is necessary for protein–protein interactions and DNA-binding. Three MYB TFs, DIVARICATA (DIV), RADIALIS (RAD), and DIV-and-RAD-interacting-factors (DRIF), compose the core DDR module for (a)symmetry establishment along the dorsal–ventral (abaxial–adaxial) axis [27] (Figure 3c). DIV and DRIF form heterodimers in the ventral region and activate unknown downstream target genes to promote ventralization. In the dorsal and lateral domains, the formation of heterodimers between DIV and DRIF is inhibited (despite their active expression) by a third MYB player, RAD, which directly binds to DRIF preventing the latter from interacting with DIV (Figure 3c). Thus, ventralization is inhibited in the dorsal and lateral regions. Accordingly, a reversion to radial symmetry and a loss of dorsal identity is observed when RAD function is lost [29]. Consistently, further studies revealed the expression of *RAD* is directly controlled by CYC and DICH, which are expressed in the dorsal region [24,30]. CYC and DICH were shown to directly bind and activate *RAD* expression [31,32], thus regulating the dorsoventral axis and flower bilateral symmetry. Therefore, controlling *CYC* and *DICH* expression is key in patterning flower bilateral symmetry. However, their upstream regulation might be more complex than anticipated and tailored in an organ-specific fashion: there are studies showing *CYC*-like expression is active in organs other than petals, such as stamens, where CYC expression expands in the lateral and ventral domains [33–35].

However, how the expression and activity of *CYC* and *DICH* are regulated remains unclear. A recent study showed the 5′ untranslated region of both *CYC* and *DICH* is significantly enriched in autoregulatory sites, which suggests a role for direct transcriptional autoregulation [36].

MADS box TFs, which play pivotal roles in determining flower organ identity in both *Arabidopsis* and snapdragon, also control flower symmetry in zygomorphic orchid flowers. Careful expression studies have shown the precise spatio-temporal pattern of MADS gene expression during orchid flower development, defining their role in specifying organ identity [31,37]. However, how the downstream MADS box TFs regulate flower symmetry patterns still requires further investigation, as do other molecular pathways underlining floral symmetry establishment in several other species [27].

## Transition in floral symmetry

Body symmetry transition and breaking events are not rare in metazoan, e.g. during echinoderm development, in which a bilaterally symmetric embryonic larvae becomes a radially symmetry adult [38], or during embryogenesis of flatfishes, where migration of one eye to the contralateral side of the skull breaks bilateral symmetry [39]. Similarly in Angiosperms, although a large proportion of flowers show consistent symmetry patterns during floral development, many experience symmetry transition events during ontogenesis, e.g. mono-symmetric, bilateral flowers may be radially symmetric in early phases, or poly-symmetric flowers may display mono-symmetry or even be asymmetric at early phases.

In early stages of *Couroupita guianensis* flower development, growth of the upper side of the flower (dorsal domain) is retarded, causing a polarisation event that engrains bilateral symmetry. This is followed by the establishment of radial symmetry when the stamen and carpel are initiated; however, at a later stage, the androecium positioned in the lower section (ventral domain) proliferates to such an extent that it forms a tongue-like structure, thus turning the developing flower into a bilaterally symmetric structure again [6,40].

Transitions in symmetry have been attributed to gradients in developmental signals along the inflorescence, leading to differential cellular/tissue growth and/or mechanical constraints resulting from neighbouring organs [6]. Also, environmental factors, such as the direction of light and gravity have been shown to influence flower symmetry transition [6,36,41,42]. It has long been known that gravity can affect the orientation of the apical–basal axis of multicellular organisms, as in brown algae of the genus *Fucus*, in which gravity helps the fertilised egg cell to fix polarity, distribute organelles asymmetrically and establish the apical–basal axis [1]. In angiosperms, gravity affects the angular position of petals in some species of zygomorphic *Saxifraga* flower, as demonstrated by experiments carried out in the absence of directional gravity resulting in the development of actinomorphic flowers [43]. While the underlining molecular mechanism connecting gravity to flower morphology is still unresolved, recent work in *Campanulaceae* found that *CYC* gene expression is preserved along the dorsal–ventral axis of a flower even as it turns upside-down, suggesting that at least late *CYC* gene expression is not regulated by gravity [44].

## Symmetry within floral organs: the gynoecium case

The fundamental importance of corolla development has been the focus of studies on flower symmetry. Sepals, petals, stamens and carpels are all modified leaves, which have acquired an astonishing variety of shapes and functions during evolution, and together they preside over plant reproduction [45]. Although fundamental for morphology and function, the understanding of symmetry establishment within individual flower organs has remained more enigmatic. In recent years, studies focused on the development of the gynoecium in several plant species have partially addressed this gap in knowledge.

In angiosperms, the carpel — the basic unit of the gynoecium — surrounds the ovules to protect them from herbivory, mechanical damage and the environment [45,46]. The carpel also ensures correct fruit development after fertilisation, and supports the release of the seeds at maturation [47,48]. Despite the astonishing morphological diversity in carpel shapes across angiosperm species, the basic organisation of the gynoecium consistently composes three parts along the basal–apical axis: the ovary, the style, and the stigma [49]. Pollen deposits on the stigma and germinates producing a pollen tube that grows internally through the style to fertilise the ovules contained in the ovary below.

**Figure 4.**
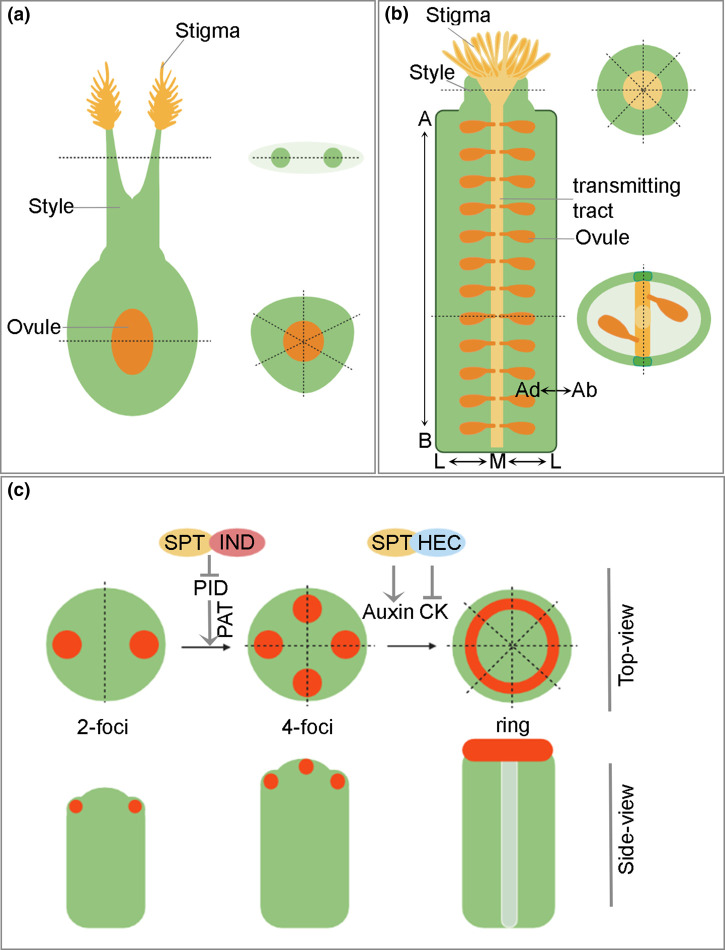
Symmetry patterning within the gynoecium of monocots and dicots. Schematic illustration of fully developed gynoecium representative of (**a**) monocots (*Oryza sativa*) and (**b**) dicots (*Arabidopsis thaliana*). (**a**) In grasses such as rice, only one ovule develops in the ovary, which is formed by three carpels, i.e. radial symmetry, while the apical branched stigma displays bilateral symmetry. (**b**) In *Arabidopsis*, cross sections in the ovary and style regions display bilateral and radial symmetry, respectively. (**c**) Schematic top- and side-views of a developing *Arabidopsis* gynoecium showing the molecular basis underpinning symmetry transition. Auxin signalling maximum (red dots) transition from 2-foci (bilateral symmetry) to 4-foci (biradial symmetry) to a ring stage (radial symmetry), during organ growth, coordinating symmetry establishment and transition with the body axes. SPATULA (SPT), INDEHISCENT (IND) and HECATEs (HECs) inhibit polar auxin transport (PAT) by repressing the kinase PINOID (PID), ultimately regulating the auxin–cytokinin (CK) balance. Illustrations are created with Biorender.com.

Interestingly, many monocots and dicots display opposite symmetry types at the end of their gynoecia. Style/stigma are bilaterally symmetric in grasses, such as rice and wheat, while the ovary is formed by the fusion of three carpel primordia, e.g. three-fold radial symmetry (Figure 4a) [50,51]. Conversely, in the dicot model plant *Arabidopsis thaliana* the gynoecium consists of two carpels congenitally fused along their marginal tissues, which grow as a tube harbouring two rows of ovules inside, and displays bilateral symmetry in cross-section along the medio-lateral axis (left–right) (Figure 4b) [52,53]. At the apical end, the *Arabidopsis* bilateral ovary gives rise to a solid structure, the style, which terminally fuses the two carpels and displays radial symmetry. Style and stigma form towards the end of the patterning phase of gynoecium development, thus a transition in symmetry, from bilateral to radial, takes place during a specific developmental window and at the distal–apical end of the growing organ. This fascinating process is controlled by key TFs and hormonal dynamic cross-talks [54]. The phytohormone auxin — whose levels are finely regulated to orchestrate plant development [55] — plays a central role in the bilateral-to-radial gynoecium symmetry transition.

Genetic and molecular studies carried out in *Arabidopsis* and rice (*Oryza sativa*) revealed comparable activity for auxin in setting up symmetry at their gynoecium apices. During gynoecium ontogenesis in *Arabidopsis*, auxin accumulates at two apical lateral foci, with a single auxin maximum at the apex of each carpel, thereby sustaining equal growth and patterning bilateral symmetry within the growing organ [56]. Subsequently, two new auxin maxima peak in the medial–apical cells to trigger the *ex novo* apical fusion. This four-foci stage of auxin distribution imposes a bi-radial state in the organ that patterns the medio-lateral axis [57,58]. Lastly, the four auxin foci merge into a ring, patterning radial symmetry at the gynoecium apex, that causes apical cells to build the style and stigma (Figure 4c) [57,59]. Several TFs, belonging to the bHLH and B3-domain families, such as SPATULA (SPT), INDEHISCENT (IND), HECATEs (HEC1,2,3) and NGATHAs (NGA1,2,3,4), play fundamental roles in this process by controlling auxin transport and biosynthesis [46,60,61]. Among these, SPT has been shown to orchestrate symmetry establishment and transition at the gynoecium apex by regulating the expression of the kinase PINOID (PID), which controls the distribution of the polar auxin transporters (PINs) [59]. In *Arabidopsis*, SPT and IND repress *PID* expression at the gynoecium apex when radial symmetry is established [59]. PID-mediated phosphorylation of PINs controls the polarity of auxin transporters and thus the flux and accumulation of the hormone. Down-regulation of *PID* causes the apolar distribution of PINs at the plasma membrane of apical cells undergoing symmetry transition, thus scaling up symmetry from the cellular to the organ level [59]. On the other hand, *PID* expression and activity has been shown to support the development of the bilateral stigma in rice, since the *pid* loss-of-function mutant lack stigmatic tissue (leading to a failure to pattern bilateral symmetry), while overexpression of *PID* in rice led to an increase in stigma development by augmenting its branching [53].

In addition, SPT also controls auxin biosynthesis and represses the cell-proliferation signal mediated by another phytohormone, cytokinin (CK), which is known to work antagonistically with auxin during plant development [57,58,62]. Moreover, SPT represses the expression of marginal *CUP-SHAPE COTYLEDON* genes (*CUC1* and *CUC2*) at the gynoecium apex [63]. CUCs are fundamental growth regulators that repress growth cell-autonomously to create boundaries and separate organs, for example during bilateral symmetry establishment in plant embryogenesis when the cotyledons form [64,65]. Altogether, the default bilateral symmetry of the *Arabidopsis* ovary results from the fusion of the two carpels at their margins and requires the activation of a dedicated genetic programme to drive radial symmetry establishment at the apical end, thus build the style and stigma of the gynoecium.

In conclusion, the genetic programme orchestrating gynoecium morphology and symmetry may be conserved and differentially regulated across angiosperms, (e.g. eudicots vs monocots) to shape their apical ends according to function.

## Summary

- Flower symmetry is significant not only in plant biology, but more broadly for co-evolution of plants and animals and their connected ecological traits. Thus, it is a theme that brings together palaeobotanists, developmental geneticists, entomologists, bioinformaticians and comparative evolutionary biologists with the focus of understanding the basis of geometrical features in biology.
- Core, conserved molecular mechanisms are co-ordinated with the establishment of the body axes and tissue patterning to set up bilateral and radial symmetry in flowers across plant species. Expanding the investigation of symmetry establishment and transition in individual flower organs is a powerful strategy to reveal novel, conserved players and biological rules underpinning symmetry during organogenesis.
- We are still far from fully deciphering how key TFs and chemical gradients orchestrate flower symmetry. With the technological advances of next-generation whole genome sequencing, powerful genetic manipulation tools (e.g. CRISPR), and phenotypical analysis, we are a step closer to drawing a large-scale picture of the molecular networks regulating flower symmetry.

## Open Access

Open access for this article was enabled by the participation of John Innes Centre in an all-inclusive *Read & Publish* agreement with Portland Press and the Biochemical Society under a transformative agreement with JISC.

## Abbreviations

CYC: CYCLOIDEA
DICH: DICHOTOMA
DIV: DIVARICATA
DRIF: DIV-and-RAD-interacting-factors
HEC1,2,3: HECATEs
IND: INDEHISCENT
PID: PINOID
PINs: polar auxin transporters
RAD: RADIALIS
SPT: SPATULA
TCP: BRANCHED1–CYCLOIDEA–PCF
